# Omnipresence of inflammasome activities in inflammatory bone diseases

**DOI:** 10.1007/s00281-019-00753-4

**Published:** 2019-09-13

**Authors:** Yael Alippe, Gabriel Mbalaviele

**Affiliations:** Division of Bone and Mineral Diseases, Washington University School of Medicine, 660 South Euclid Avenue, Campus Box 8301, St. Louis, MO 63110 USA

**Keywords:** Inflammation, Inflammasomes, Osteoclasts, Bone resorption, NLRP3, Cytokines

## Abstract

The inflammasomes are intracellular protein complexes that are assembled in response to a variety of perturbations including infections and injuries. Failure of the inflammasomes to rapidly clear the insults or restore tissue homeostasis can result in chronic inflammation. Recurring inflammation is also provoked by mutations that cause the constitutive assembly of the components of these protein platforms. Evidence suggests that chronic inflammation is a shared mechanism in bone loss associated with aging, dysregulated metabolism, autoinflammatory, and autoimmune diseases. Mechanistically, inflammatory mediators promote bone resorption while suppressing bone formation, an imbalance which over time leads to bone loss and increased fracture risk. Thus, while acute inflammation is important for the maintenance of bone integrity, its chronic state damages this tissue. In this review, we discuss the role of the inflammasomes in inflammation-induced osteolysis.

## Introduction

**Fig. 1:**
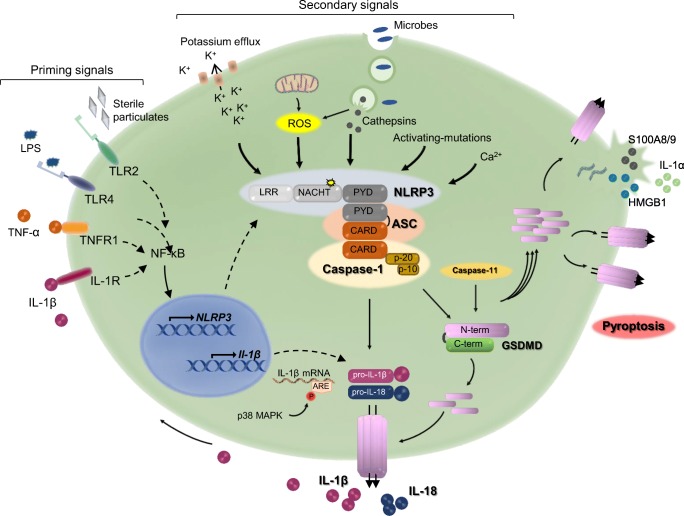
**Mechanisms of activation of the NLRP3 inflammasome.** Activation of the NLRP3 inflammasome involves two steps. Induction of priming signals upon ligation of pathogen recognition receptors (PRRs) such as TLR4 and TLR2, and cytokine receptors including IL-1 receptor (IL-1R; positive feedback) and TNF receptor (TNFR). These signals induce the transcription of NLRP3 and pro-IL-1β through NF-κB; pro-IL-18 is constitutively expressed. Priming of NLRP3 can also be induced by its deubiquitination, independently of de novo protein synthesis (not depicted). pro-IL-1β mRNA are stabilized by p38α MAPK. Increased expression of NLRP3 enables the recruitment of pro-caspase-1 via ASC upon sensing of secondary signals, which are triggered by a wide range of stimuli including K^+^ efflux, Ca^2+^ influx, phagocytosis of microorganisms, and particulate materials (causing lysosome destabilization/rupture and release of cathepsins and reactive oxygen species (ROS)), and mitochondrial dysfunction. *NLRP3*-activating mutations in the NACHT domain cause constitutive activation of this inflammasome. Proximity-induced reaction leads to auto-activation of caspase-1, which then processes pro-IL-1β and pro-IL-18 into IL-1β and IL-18, respectively. Caspase-1 also cleaves GSDMD into GSDMD-N-terminal (N-term) and GSDMD-C-terminal (C-term) fragments. GSDMD-N-term translocates to the plasma membranes where it oligomerizes and forms pores through which IL-1β and IL-18 are secreted. However, excessive pore formation causes pyroptosis, resulting in the release of not only IL-1β and IL-18, but also other mediators such as IL-1α, S100A8/9, and HMGB1. ARE, AU rich elements; ASC, apoptosis-associated speck-like protein containing a CARD; GSDMD, gasdermin D; HMGB1, high-mobility group box 1; IL-1, interleukin-1; LPS, lipopolysaccharide; NF-κB, nuclear factor kappa B; NLRP3, NLR family, pyrin domain containing 3; TLR, toll-like receptor; TNF-α, tumor necrosis factor-α. Stimulation of the noncanonical NLRP3 inflammasome also occurs secondarily to the activation of caspase-11, which also cleaves GSDMD

Nucleotide-binding oligomerization domain-like receptors (NLRs, e.g., NLRP1) or absent in melanoma 2-like receptors (ALRs, e.g., AIM 2) associate with caspase-1 directly or indirectly via apoptosis-associated speck-like protein containing a CARD (ASC) to form intracellular protein complexes called inflammasomes. These macromolecular structures are assembled in response to perturbations caused by microbial products also known as pathogen-associated molecular patterns (PAMPs). For example, anthrax lethal factor, bacterial muramyl dipeptide, and bacterial flagellin induce the nucleation of the NLRP1 inflammasome, NLRP3 inflammasome, and NLRC4 inflammasome, respectively [1, 2]. The inflammasomes are also activated by host endogenous cues from damaged cells or exogenous materials, signals commonly known as danger-associated molecular patterns (DAMPs). In this regard, the NLRP3 inflammasome stands out, owing to its ability to sense a wide range of structurally different molecular entities including crystalline materials, misfolded or aggregated proteins, metabolites, prosthetic implant wear debris, and certain materials found in the environment such as asbestos and silica particles [3–5] (Fig. 1). The NLRC4 inflammasome and AIM2 inflammasome are also activated to some extent by endogenous DAMPs [6–8]. The inflammatory responses induced by PAMPs or DAMPs can be either acute when the perturbation is rapidly resolved, and the homeostasis is restored, or chronic and pathologic when rapid clearance mechanisms fail. Finally, activating mutations in *NLRP1*, *NLRP3*, *NLRC4*, or *MEFV* cause inflammasome assembly independently of PAMPs or DAMPs [9–15].

Caspase-1 processes pro-interleukin-1β (pro-IL-1β) and pro-IL-18 into IL-1β and IL-18, respectively [16]. It also cleaves gasdermin D (GSDMD), generating an N-terminal fragment that translocates from the cytoplasm to the plasma membrane where it forms pores through which IL-1β and IL-18 are secreted [17, 18]. However, excessive pore formation resulting from sustained activation of GSDMD in both infectious and sterile conditions compromises membrane integrity, and ultimately ruptures the cell, releasing pro-inflammatory cytoplasmic contents into the extracellular environment. This form of cell death, termed pyroptosis, is inflammatory and results in the recruitment of immune cells and the perpetuation of inflammation [19, 20]. Caspase-8 and neutrophil elastase can also generate IL-1β and IL-18 whereas caspase-8, caspase-11 (ortholog of human caspase-4 and caspase-5), and neutrophil elastase efficiently process GSDMD [21–28]. Sustained exposure to supra-physiological levels of IL-1β and IL-18, ultimately, inflicts damage to multiple tissues including the skeleton.

Coordinated actions of the osteoclasts and the osteoblasts are essential to maintain bone mass and quality. The osteoclasts remove the old or defective matrix, which is replenished fully by the osteoblasts; this tightly regulated process is known as bone coupling [29]. Several growth factors, including bone morphogenetic proteins (BMPs) and Wnts control the differentiation of the osteoblasts from mesenchymal stem cells whereas the osteoclasts differentiate from myeloid progenitors exposed to signals generated by macrophage colony-stimulating factor (M-CSF) and receptor activator of NF-κB ligand (RANKL) [30, 31]. The osteoblast and osteoclast differentiation programs are antagonized and enhanced, respectively, by pro-inflammatory cytokines such as tumor necrosis factor-α (TNF-α) and IL-1β. Excessive bone resorption by the osteoclasts at the expenses of bone formation by the osteoblasts in inflammatory conditions creates an imbalance, which over time leads to bone loss and increased fracture risk [32]. In this article, we review the role of the inflammasomes in bone resorption and highlight the collateral effects of these protein complexes on other skeletal cells.

## Mechanisms of bone resorption that are relevant to the inflammasomes

IL-1β plays numerous roles in bone pathophysiology. It stimulates RANKL production by mesenchymal cells (e.g., stromal cells, osteoblasts, and osteocytes), synoviocytes, and T cells directly or indirectly through the regulation of IL-6, IL-17, and TNF-α expression [33–38]. The reciprocal regulation of IL-1β production by these cytokines and the reported RANKL-independent actions of IL-6 and TNF-α in osteoclast differentiation indicate that these responses are complex and multidirectional [39, 40]. Irrespective of the hierarchy of the events, IL-1β and its effectors act in synergy with RANKL to promote osteoclast differentiation and activity while suppressing osteogenesis [32]. IL-1β also stimulates its own synthesis, a positive feedback mechanism that underlies the chronicity of inflammasome actions in bone diseases [33, 41]. On the other hand, while pro-inflammatory actions of IL-18 in skin disorders [42] and infection-associated allergic diseases [43] are well described, the role of this cytokine in bone is ambiguous. Indeed, IL-18 can inhibit or stimulate bone resorption, depending on cell-contexts [44–46].

Secretion of IL-1β through GSDMD-assembled pores by live cells has been reported [18, 26]. This phenomenon referred to as a hyper-reactive state occurs independently of pyroptosis and may be characteristic of diseases of low-grade inflammation as discussed later in this review. GSDMD pores have an inner diameter of 10–20 nm, which is much bigger than the diameter of mature IL-1β and IL-18 (approximately 5 nm) [17, 20, 47–49]. There is thus far no basis for such large pores to facilitate only the secretion of these cytokines, implying that molecules with smaller sizes such as eicosanoids, which are also important regulators of bone resorption may be secreted through GSDMD pores. On the other hand, pyroptosis is presumably prominent in diseases marked by chronic episodes of high-grade inflammation such as inflammasomopathies. In this context, IL-1β and IL-18 are concomitantly secreted alongside alarmins including IL-1α, S100A8/9, HMGB1 [50, 51], and possibly lipid mediators such as eicosanoids [52]; this outcome may underlie the limited efficacy of IL-1 blockers in the treatment of bone diseases. Indeed, not only are these alarmins produced by myeloid cells, synovial cells, and osteoblasts [53, 54], but they are also potent modulators of inflammation and osteoclastogenesis, regulating the expression of RANKL, TNF-α, IL-1β, and IL-6 [33, 55]. Inflammasome signaling also leads to the cleavage of poly(ADP-ribose) polymerase 1 (PARP1) by caspase-7, a response that ultimately promotes the degradation of this negative regulator of osteoclast differentiation and bone resorption [56]. Thus, the inflammasomes are key players in the pathogenesis of inflammatory osteolysis. Understanding the biology of these signaling platforms is essential for the development of effective therapies targeting inflammatory bone loss.

## Widespread activities of the inflammasomes in inflammatory osteolysis

Evidence suggests that the inflammasomes are implicated in a wide range of diseases of bone loss driven by sterile and non-sterile inflammation (Table 1).

**Table 1:** Inflammasomes and their activators in inflammatory bone diseases

Activators	Disorders	Description of the activators	Inflammasomes
PAMPs	Periodontitis^[[57–59^^]^	*Porphyromonas gingivalis*	NLRP3, AIM2
	Osteomyelitis^[[60–62^^]^	*Staphylococcus aureus*	NLRP3
Mutations	CAPS^[[63–79^^]^	Autosomal dominant	NLRP3, NLRC4, NLRP12
	MAS^[[11, 12, 80, 81^^]^	Autosomal dominant	NLRC4
	FMF^[[82, 83^^]^	Autosomal recessive	Pyrin
DAMPs	Sterile CRMO^[[22, 23^^]^	Unknown	NLRP3
	Arthritis^[[84–88^^]^	Self-DNA, other DAMPs?	NLRP3, AIM2, others?
	Metabolic diseases, aging^[[5–8, 89–91^^]^	Purine metabolites, fatty acids, other DAMPs?	NLRP3, NLRC4, AIM2, others?
	Wear debris osteolysis^[[3, 92, 93^^]^	PMMA, CoCrMo, etc.	NLRP3, AIM2
	Crystal-induced arthropathies^[[94–97^^]^	MSU crystals (gout), CPPD crystals (pseudogout), BCP crystals	NLRP3

### Cryopyrin-associated periodic syndromes

Cryopyrin-associated periodic syndromes (CAPS), which include familial cold autoinflammatory syndrome (FCAS), Muckle–Wells syndrome (MWS), and neonatal-onset multisystem inflammatory disease (NOMID) are caused by autosomal dominant mutations in the NACHT domain of NLRP3 [63–65, 98]. Additionally, myeloid-restricted somatic mosaicism and mutations in *NLRP12* and *NLRC4* may account for the inflammatory responses in CAPS patients negative for mutations in *NLRP3* [66]. NLRP3 is believed to switch from a closed and inactive conformation to an open state in response to PAMP- or DAMP-induced cues; *NLRP3*-activating mutations locked this protein in the active state, leading to the constitutive assembly of the inflammasome [67]. Common features of CAPS include recurring fever episodes, urticaria, conjunctivitis, and joint pain whereas central nervous system complications and arthropathies characterized by bone deformities, bulky epiphyses, leg length discrepancy, and short stature are hallmarks of NOMID, the most severe manifestation of these disorders [66, 68–70]. Consistent with the tumor-like features of bony outgrowths, induced pluripotent stem cells from NOMID patients are more proliferative and exhibit higher differentiation potential than normal cells [71]. Epiphyseal abnormalities undoubtedly predispose NOMID patients to joint instability and subsequent development of osteoarthritis. It is worth noting that skeletal phenotyping is based on radiographic observations and limited histology that reveal heterogeneously calcified bone matrix, and severely disorganized and hypocellular growth plate [70].

Murine models of CAPS in which wild-type *Nlrp3* alleles are replaced by murine or human alleles carrying mutations found in patients, reproduce several features of human disorders including early onset of systemic inflammation, skin and joint pathologies, and growth retardation. Cryopyrinopathies are in general more severe in rodents than in humans as mutant mice of all phenotypes (i.e., CAPS, MWS, and NOMID) exhibit short lifespan (3–4 weeks) [72–74]. NOMID mice in which NLRP3 is activated globally exhibit normal patterning of skeletal elements but display hypocellular epiphyses due to massive chondrocyte death, and disorganized growth plate matrix protruding towards the bone marrow cavity [73]. Unexpectedly, conditional activation of NLRP3 in myeloid cells but not in osteochondro-progenitors reproduces the abnormal cartilage features, suggesting that the phenotype is not chondrocyte autonomous [75]. CAPS mice also exhibit severe low bone mass, a phenotype that correlates with a massive expansion of osteoclast precursors, exuberant osteoclastogenesis, and increased osteoclast activity [41, 73, 75–77]. Thus, while the magnitude of bone resorption in CAPS patients is not known, this process is prominent and well characterized in mouse models.

Systemic inflammation and multiple organ pathologies, including bone abnormalities, are entirely prevented in NOMID mice lacking IL-1 receptor [75]. However, persistent residual inflammation is reported in FCAS mice and MWS mice deficient in IL-1 and IL-18 signaling [78, 79]. These findings align with clinical studies, which consistently show that epiphyseal lesions and outgrowths continue to expand for a significant number of NOMID patients on IL-1 biologics despite the resolution of disease-associated inflammatory symptoms [70, 99–101]. Collectively, these observations suggest that IL-1β is not the primary driver of skeletal outcomes in CAPS, and that inflammasome-dependent responses other than IL-1β play a role in these disorders. This view is consistent with findings showing that levels of TNF-α remain elevated in certain CAPS patients on IL-1 blockers, and neutralization of TNF-α activity improves inflammatory endpoints in CAPS mice [78]. This view is further supported by recent evidence indicating that the pathogenesis of NOMID in mice is prevented by (i) genetic ablation of GSDMD and (ii) a novel inhibitor of the interactions of p38α MAPK and MAPK-activated kinase 2, which inhibits not only IL-1β, but also IL-6 and TNF-α [41, 77]. Thus, multiple responses, including pyroptosis, contribute to inflammasomopathies.

### Macrophage activation syndrome

The NLRC4 inflammasome senses bacterial type III and IV secretion systems and flagellin via NLR family apoptosis inhibitory proteins (NAIPs). As noted above, *NLRC4*-activating mutations are found in certain FCAS patients. Moreover, recent evidence implicates the NLRC4 inflammasome in the pathogenesis of sterile inflammatory disorders as patients with *NLRC4* gain-of-function mutations develop cytopenia, high ferritin levels, hemophagocytosis, and splenomegaly. These symptoms are associated with excessive levels of IL-18 and IL-1β, and recurring fever flares, a phenotype that is reminiscent of macrophage activation syndrome (MAS) [12, 80]. MAS is a frequent complication of systemic juvenile idiopathic arthritis (sJIA), a disease that interferes with healthy skeletal development and bone mass acquisition [102]. Although IL-1β levels are in general lower in MAS compared with CAPS, IL-1 biologics are efficacious in the treatment of sJIA [103, 104]. Consistent with the role of mutated NLRC4 in the development of joint pathologies, transgenic mice expressing constitutively active NLRC4 produce high levels of IL-1β and IL-18, and develop arthritis [81]. The NLRC4 inflammasome is also activated by nucleotide-derived metabolites (e.g., adenine and N-4-acetylcytidine) [6] and fatty acids (e.g., lysophosphatidylcholine and palmitate) [89] and is upregulated by bone-derived DAMPs during osteoclastogenesis [90]. An interplay between the NLRP3 and NLRC4 inflammasomes has been noted in these models of sterile inflammation as well as in response to *Salmonella* infection [105, 106].

### Familial Mediterranean fever

*MEFV* encodes pyrin, which activates caspase-1 through ASC upon sensing post-translationally modified Rho GTPase [107]. These modifications include phosphorylation, ADP-ribosylation, and glycosylation and occur upon cell exposure to *Clostridium* toxins and in conditions of mevalonate kinase deficiency or proline-serine-threonine phosphatase-interacting protein 1 (PSTPIP1) gain-of-function [82, 108–110]. Hyper-activation of the pyrin inflammasome by *MEFV*-activating mutations causes Familial Mediterranean fever (FMF), a disease that is characterized by high levels of IL-1β, IL-6, IL-8, and IL-12, recurring fever episodes, arthritis, and low bone mass [83, 111]. FMF is the most prevalent monogenic autoinflammatory disorder; it affects over 100,000 persons worldwide and causes sporadic and chronic symptoms [98]. FMF mice develop severe systemic inflammation and exhibit massive cartilage and bone erosion [112]. These mice do not display systemic inflammatory symptoms upon deletion of GSDMD, IL-1 receptor, or ASC [112, 113].

### Arthritis

Inflammasomes are activated in several autoimmune diseases, including rheumatoid arthritis (RA) and ankylosing spondylitis (AS). Components of the NLRP3 inflammasomes and various cytokines including TNF-α, IL-1β, IL-6, IL-7, and IL-17 are highly expressed in RA [84, 114]. Specific interactions among these cytokines and other inflammatory mediators may drive systemic and focal osteolysis in arthritis [115]. Systemic arthritis and bone loss induced by TNF over-expression are abolished upon ablation of IL-1β signaling despite the presence of synovial inflammation, suggesting that the effects of TNF-α on bone are mediated by IL-1β [116]. These findings positioning IL-1β downstream of TNF-α are in line with the observation that TNF-α-induced RANKL production by murine stromal cells is dependent on IL-1β [85, 117]. As noted above, the upregulation of TNF-α by IL-1β is also well known. Components of the inflammasomes, including NLRP3, ASC, and caspase-1 are upregulated in AS, a disease that is also associated with high levels of IL-1β, TNF-α, IL-6, IL-23, and IL-17 [118]. Bone manifestations in AS include excessive focal bone formation in joints whereas pronounced trabecular bone loss occurs in vertebral bodies [119].

Various inflammasomes are assembled in mouse models of arthritis. For example, NLRP3 and AIM-2 are both upregulated in the synovium of IL-10-deficient mice exposed to antigen-induced arthritis, and osteoclast differentiation from bone marrow cells isolated from these mutant mice is blunted by the inhibitors of NLRP3 and AIM-2 inflammasomes [120]. Moreover, arthritis induced by DNase II deficiency, which is associated with accrual of self-DNA, is attenuated by AIM2 ablation [86, 87]. The complexity of inflammasome functions is underscored by the observations that NLRP3, NLRP1, NLRC4, and caspase-1, but not ASC are dispensable for collagen-induced and antigen-induced arthritis [88, 121]; yet NLRP3 is a key player in joint destruction in a mouse model of A20 deficiency, in which NLRC4 and AIM2 are expendable [122]. Thus, the role of the inflammasomes in experimental arthritis is mouse model-dependent.

### Osteomyelitis

Defective innate immune defense mechanisms including the inflammasomes underlie the pathogenesis of periodontitis and osteomyelitis commonly caused by *Porphyromonas gingivalis* and *Staphylococcus aureus*, respectively. *P. gingivalis*–derived PAMPs such as LPS are potent activators of priming signals, which through TLR4 signaling drive the expression of several components of the inflammasomes including IL-1β, NLRP3, AIM2, and caspase-11 [57, 58]. Accordingly, the massive alveolar bone destruction caused by *P. gingivalis* is attenuated upon loss of NLRP3 [59]. *S. aureus* bacterial products include peptidoglycans, hemolysins, bacterial lipoproteins, and Panton-Valentine leucocidin stimulate the inflammasomes through TLR2-mediated activation of NF-kB [123]. Moreover, some of these factors promote osteoclastogenesis [124]. The osteoblasts also express the NLRP3 inflammasome [60] though to lower levels compared with myeloid cells [73] and contribute to the pathogenesis of periodontitis and osteomyelitis [61, 125–127].

Autoinflammatory reactions of unknown etiology causes confined chronic non-bacterial osteomyelitis (CNO) or systemic chronic recurrent multifocal osteomyelitis (CRMO). Components of the NLRP3 inflammasome are expressed in osteoclasts in bone specimens from CRMO patients [62]. Mice carrying an inactivating -mutation in the proline-serine-threonine phosphatase-interacting protein 2 gene (*Pstpip2*) develop a phenotype reminiscent of CRMO, which is associated with over-production of IL-1β, enhanced osteoclastogenesis and bone resorption, responses that depend on IL-1 receptor and IL-1β, but not IL-1α, and are driven by neutrophils [21, 22]. Neutrophils in CRMO mice over-produce IL-1β via redundant actions of caspase-8 and the NLRP3 inflammasome [23].

### Metabolic bone diseases

Inflammasomes have been linked to age- and menopause-related osteoporosis [6, 90, 91]. Estrogen profoundly affects the skeleton through various mechanisms including immunomodulation, suppressive effects on the expression of TNF-α and IL-1β, induction of osteoclast apoptosis through ERα, suppression of osteoclast differentiation, inhibition of RANKL production by osteoblasts, T and B cells, and stimulation of osteoprotegerin (OPG) expression [31, 128, 129]. Consistent with increased levels of pro-inflammatory cytokines in conditions of estrogen deficiency, blockade of TNF-α or IL-1β in post-menopausal patients leads to a decrease in the levels of bone resorption markers [130]. Accordingly, inhibition of TNF-α or deletion of NLRP3 protects against ovariectomy-induced bone loss in mice [90, 131].

Aging is associated with low-grade chronic inflammation. This process is referred to as inflammaging and is associated with increased levels of circulating IL-18, IL-1 receptor antagonist, and IL-6 [132]. Inflammasome gene modules including NLRC4 and IL-1β are upregulated in older people compared with younger individuals; persistent expression of these genes correlates with the occurrence of age-related complications, including chronic production of inflammatory cytokines, metabolic dysfunction, and oxidative stress [6]. The NLRP3 inflammasome also modulates age-related inflammation in peripheral tissues and age-related bone loss in mice, though the underlying mechanisms are unknown [91].

The ability of the NLRP3 inflammasome to detect a wide variety of endogenous DAMPs is likely an important driver in the development of age- and metabolic-related pathologies. These DAMPs include crystalline cholesterol, extracellular ATP, purine and pyrimidine metabolites, and debris from damaged tissues [6, 133]. For example, metabolites from the purine and pyrimidine pathways stimulate the NLRP3 and NLRC4 inflammasomes in THP-1 cells, activate human platelets and neutrophils in cultures, and promote hypertension and inflammation in mice [6]. The NLRP3 inflammasome may also be involved in hyper-multinucleation of murine osteoclasts caused by purinergic receptor P2X5 signaling [134]. We have shown that bone matrix degradation products regulate the NLRP3 and NLRC4 inflammasomes in cells of the osteoclast lineage [90]. Accordingly, *Nlrp3* null mice are protected from bone loss induced by ovariectomy, sustained exposure to parathyroid hormone or RANKL. Treatment of mice with zoledronic acid inhibits inflammasome activation, thus reinforcing the view that endogenous DAMPs are released from the bone matrix during bone resorption, causing autocrine and paracrine effects on osteoclastogenesis [90].

### Wear debris-induced osteolysis

Wear particles from articulating prosthetic joint surfaces such as those from cobalt-chromium-molybdenum (CoCrMo) implants induce inflammatory responses that cause aseptic loosening as a result of uncontrolled osteolysis [135]. The osteolytic process is associated with the formation at the implant-bone interface of a cellular membrane enriched in cells of the monocyte-macrophage lineage [136]. Activation of the NF-kB pathway through TLR2 signaling by prosthetic debris promote not only the expression of pro-inflammatory cytokines such as TNF-α, but also priming signals for the NLRP3 and AIM2 inflammasomes. Macrophages can also phagocytose these particles; cellular accumulation of these non-degradable materials enhances the production of reactive oxygen species and the rupture of the phagosomes, which releases cathepsins in the cytoplasm, events that activate the inflammasomes [92]. The size and shape of CoCrMo alloys affect the amplitude of the inflammatory responses [93]. Thus, wear debris can provide both priming and assembly signals that lead to aberrant inflammasome activation. Consistent with the role of the NLRP3 inflammasome in bone damage induced by prosthetic particles, bone resorption induced by polymethylmethacrylate (PMMA) particles is reduced in the absence of caspase-1 [3, 145]. Thus, both the metal and plastic components of the prostheses activate the inflammasomes.

### Crystal-induced arthropathies

Endogenous crystalline particles are involved in the pathogenesis of arthropathies. For example, gouty arthritis is caused by precipitation of monosodium urate (MSU) crystals [94], calcium pyrophosphate deposition disease (CPDD) is driven by calcium pyrophosphate dihydrate (CPPD) crystals [94], and degenerative arthropathies such as osteoarthritis and Milwaukee shoulder are the result of abnormal accumulation of basic calcium phosphate (BCP) crystals [137]. Shared mechanisms among these diseases include phagocytosis of crystals by myeloid cells, an event that activates several inflammatory pathways including the inflammasomes, chondrocyte apoptosis, and matrix calcification [138–140]. Bone erosions in gout stems from continuous recruitment of macrophages to tophi [95]; sporadic CPDD is characterized by the presence of CPPD crystals in articular cartilage whereas patients with CPDD familial patterns have low bone mineral density though the extent to which bone resorption is impacted is not known [141]. BCP crystals, particularly hydroxyapatite crystals, promote osteoclast formation in vitro in NLRP3 inflammasome-dependent manner [90, 96, 142]. However, the actions of BCP on NLRP3 inflammasome-mediated skeletal pathology are controversial. Lack of components of the NLRP3 inflammasome prevents the development of neutrophil inflammation in the air-pouch model of synovitis and decreased pathology in the Ank-deficient model of arthritis [97]. By contrast, inflammatory responses induced by the injection of BCP crystals into the knees or intra-peritoneally are independent of the NLRP3 inflammasome [143, 144]. Alternative processing of IL-1β by caspase-8 and pyroptosis may account for these discrepant observations.

## Therapeutic perspective

**Fig. 2:**
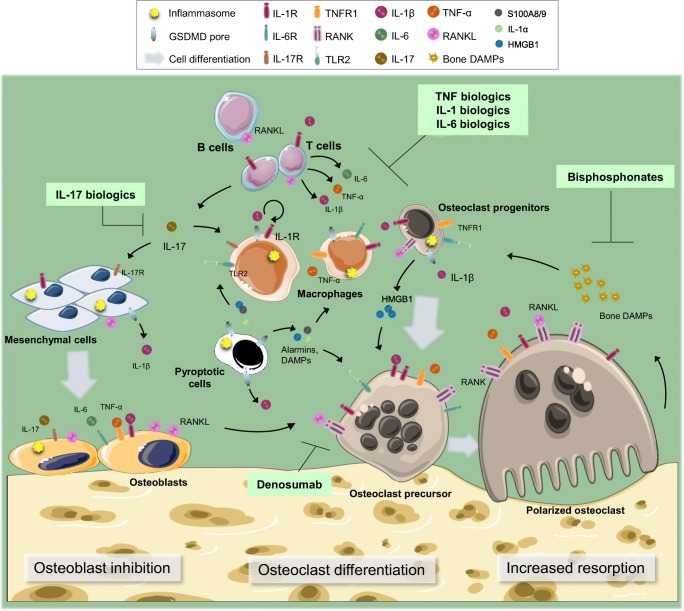
**Inflammatory osteolysis and therapeutic interventions.** Cytokines (e.g., IL-1α, IL-1β, IL-6, IL-17, and TNF-α) stimulate bone resorption directly, acting on the osteoclast lineage or indirectly by inducing RANKL expression by mesenchymal cells, T cells, and B cells. These cytokines also inhibit bone formation. Bone resorption is directly blocked by anti-resorptive drugs such as bisphosphonates and denosumab or indirectly by biologics targeting IL-1α/IL-1β, IL-6, IL-17, or TNF-α. In conditions of low-grade inflammation, bone resorption is amplified by DAMPs that are released from bone matrix

Anti-resorptive drugs such as bisphosphonates and denosumab are efficacious in the prevention of inflammation-associated bone fractures, but they do not impact the course of inflammation (Fig. 2). Thus, inhibition of osteoclast differentiation and/or activity is not sufficient to arrest the damage to the bone surrounding soft tissues such as the synovium in conditions of high-grade inflammation. On the other hand, biologics are successfully used in the clinic to temper down inflammation (Fig. 2). However, biologics have their shortcomings such as high costs, the requirement for parenteral delivery, the development of resistance, and immunosuppression. In addition, the efficacy of these drugs is restrained by redundancy among signaling pathways as they target specific inflammatory instigators. Thus, there is still an unmet medical need for the development of adequate therapeutic strategies; in-depth understanding of the mechanism of action of key inflammatory pathways is required to achieve this goal. Recent breakthroughs revealing that aberrant activities of the inflammasomes cause pyroptosis, a lytic form of cell death that concomitantly unleashes several inflammatory mediators to the extracellular milieu offer novel perspectives for drug discovery. For example, strategies aimed at preventing pyroptosis through selective blockade of individual components of the inflammasomes such as caspase-1, NLRP3, or GSDMD or inhibiting signaling nodes that integrate several inflammatory cues such as p38 MAPK are being fiercely explored.
